# Effects of different physical activity modalities on executive function in children with attention deficit hyperactivity disorder: a systematic review and meta-analysis

**DOI:** 10.3389/fpsyt.2026.1824121

**Published:** 2026-06-24

**Authors:** Changzhou Chen, Mingyuan Fan, Chuanwen Yu, Shuli Wan

**Affiliations:** 1 Shanghai University of Sport, Shanghai, China; 2 Heze University, Heze, China; 3 Shanghai Urban Construction Vocational College, Shanghai, China

**Keywords:** attention-deficit/hyperactivity disorder, cognitive flexibility, executive functions, physical activity, working memory, inhibitory control

## Abstract

**Objective:**

This study systematically evaluated and compared the effects of different physical activity modalities on executive function, including inhibitory control, cognitive flexibility, and working memory, in children with attention-deficit/hyperactivity disorder (ADHD).

**Methods:**

Six databases (CNKI, Wanfang, Web of Science, PubMed, Cochrane Library, and Embase) were searched for randomized controlled trials examining physical activity interventions for executive function in children with ADHD. Studies were screened using EndNote 21, methodological quality was assessed with the Cochrane Risk of Bias 2.0 tool, and meta-analyses were conducted in RevMan 5.4. Standardized mean differences (SMDs) with 95% confidence intervals (CIs) were calculated, and publication bias was tested using Stata 19.0.

**Results:**

Twenty-one RCTs involving 915 participants were included. Physical activity significantly improved executive function in children with ADHD, with interventions lasting at least six weeks and delivered at moderate-to-vigorous intensity showing greater overall benefits. Open physical activities produced stronger improvements in inhibitory control (SMD = -0.81, 95% CI [-0.97, -0.52], P < 0.00001), whereas closed physical activities were more effective for cognitive flexibility (SMD = -0.61, 95% CI [-0.84, -0.38], P < 0.00001) and working memory (SMD = -0.45, 95% CI [-0.68, -0.23], P < 0.0001). Single aerobic activities showed better effects on inhibitory control and cognitive flexibility, while multi-component activities were more beneficial for working memory. By activity type, leisure-fitness activities had the strongest effect on inhibitory control, traditional aerobic activities such as running and cycling were most effective for cognitive flexibility, and ball sports showed superior effects on working memory.

**Conclusion:**

Structured physical activity is an effective non-pharmacological intervention for improving executive function in children with ADHD. Interventions lasting at least six weeks at moderate-to-vigorous intensity appear most beneficial. Different modalities may target distinct executive domains: open and leisure-fitness activities for inhibitory control, closed and aerobic activities for cognitive flexibility, and closed, multi-component, and ball-sport activities for working memory.

**Systematic review registration:**

https://www.crd.york.ac.uk/PROSPERO/, identifier CRD420251080013.

## 1 Introduction

Attention-deficit/hyperactivity disorder (ADHD) is one of the most common neurodevelopmental disorders in childhood and adolescence. It is characterized by persistent patterns of inattention, hyperactivity, and impulsivity that interfere with normal functioning and development (1, 2). Epidemiological data suggest a global prevalence of approximately 7.2%, with an upward trend in recent years (3). In China, the prevalence is estimated at about 6.26% (4), with a male-to-female ratio of roughly 2.2:1 (5). Owing to its high prevalence, chronic course, and substantial impact on academic, social, and emotional functioning, ADHD has become a major public health concern, underscoring the need for effective and sustainable intervention strategies.

Executive function (EF) refers to a set of higher-order, top-down cognitive processes responsible for self-regulation, goal-directed behavior, and adaptive control of thoughts, actions, and emotions (6). Core components of EF include inhibitory control (the self-regulation of attention and behavior), working memory (the maintenance and manipulation of information), and cognitive flexibility (the ability to shift between tasks or mental sets) (7). These fundamental capacities are essential for children’s academic achievement, emotional regulation, and social adaptation (8, 9). Deficits in EF are well documented in children with ADHD and are regarded as central to its cognitive pathology.

Conventional management of EF impairments in ADHD typically adopts a multimodal approach that combines pharmacological and non-pharmacological interventions. Pharmacotherapies—primarily central nervous system stimulants (e.g., methylphenidate, amphetamines) and non-stimulants (e.g., atomoxetine)—can reduce core symptoms (10, 11). However, they are associated with side effects, potential dependence, and uncertain long-term efficacy. Behavioral therapies, although free from pharmacological risks, require prolonged implementation and substantial human and material resources (12). Consequently, there is growing interest in alternative, safe, and accessible interventions that can complement or partially substitute traditional treatments.

Physical activity (PA) has recently emerged as a promising non-pharmacological intervention for ADHD. Evidence suggests that PA may act through neurobiological mechanisms similar to those of stimulant medications, potentially enhancing dopaminergic and noradrenergic signaling and exerting synergistic effects with pharmacotherapy (13). Studies have shown that cognitively engaging physical activities can produce more substantial and lasting improvements in EF and ADHD symptoms (14). Long-term PA interventions (≥12 weeks) have been reported to enhance EF in children with ADHD (15), whereas even a single session of moderate-intensity aerobic exercise may acutely improve inhibitory control and neurocognitive performance (16). However, findings are not entirely consistent; some studies report no significant changes in inhibitory control following short-term aerobic exercise sessions (17).

Despite accumulating evidence for the beneficial effects of PA on EF in ADHD, considerable heterogeneity exists in the types, intensities, and durations of exercise interventions. Previous studies have examined diverse forms of PA, including traditional aerobic exercise (e.g., running, cycling), aquatic activities, ball games, single-mode aerobic exercise, and multi-component training. It remains unclear, however, whether different PA modalities exert distinct effects on specific EF sub-components. Moreover, most existing research has focused on general healthy populations or adults, with relatively few studies targeting school-age children with ADHD. Existing meta-analyses have tended to concentrate on a single EF subcomponent (e.g., inhibitory control) or a single type of PA (e.g., aerobic exercise), and the impact of PA intervention frequency on EF improvement remains insufficiently understood. Therefore, this meta-analysis aims to systematically examine the effects of various PA modalities on the three core dimensions of EF—inhibitory control, cognitive flexibility, and working memory—in school-age children with ADHD. In addition, it categorizes PA intervention duration, frequency, and intensity, with the goal of providing empirical evidence and theoretical guidance for optimizing future PA-based intervention programs to improve EF in this population.

## 2 Methods

This meta-analysis was performed in compliance with the statement of Preferred Reporting Items of Systematic Reviews and Meta-Analysis (PRISMA) 2020 (18). The registered ID of this study in PROSPERO is CRD420251080013.

### 2.1 Search strategy

A comprehensive literature search was conducted through databases including PubMed, The Cochrane Library, Embase, Web of Science, China National Knowledge Infrastructure (CNKI), and Wanfang Data through April 20, 2026. The aim was to identify randomized controlled trials examining the effects of physical activities on executive function among children with ADHD. The search strategy was structured around the PICOS criteria, with children with ADHD as the target population (P), different physical activities as the intervention (I), no intervention or alternative treatments as the comparator (C), and executive function including inhibitory control, working memory and cognitive flexibility as the outcomes (O), and randomized controlled trials (RCTs) as the study design (S). Two authors (Chen CZ and Fan MY) independently screened the studies. In cases of disagreement, a discussion took place with a third author until a consensus was reached.

The search strategy combined controlled vocabulary terms (e.g., MeSH and Emtree terms) with free-text keywords to maximize sensitivity and specificity. Boolean operators (“AND,” “OR,” and “NOT”) were used to combine search terms appropriately. In addition, the reference lists of all included studies and relevant reviews were manually screened to identify any potentially eligible studies that the electronic search might have missed. The detailed English-language search strategy (using PubMed as an example) is presented in Appendix **Table 1**.

**Table 1:** Main characteristics of studies included.

Study name	Country	Participant description		Intervention group				Control group	Executive function	Measures
		IG/CG	Age	Intervention type	Type	Intensity	Duration/Frequency/Week			
Chang et al., 2012 (19)	China	20/20	10.45 ± 0.95/10.42 ± 0.87	Aerobic exercise	Closed	MPA	30min*1*1week	Watch video	13	①⑤
Bustamante et al., 2016 (20)	USA	19/16	8.70 ± 2.00/9.40 ± 2.20	Structured play	Open	MVPA	90min*5*10weeks	Art project	12	⑰⑱
Pan et al., 2016 (21)	China	16/16	8.93 ± 1.49/8.87 ± 1.56	Table tennis	Open	NR	70min*2*12weeks	Wait-list	1	①
Memarmoghaddam et al., 2016 (22)	Iran	19/17	8.31 ± 1.29/8.29 ± 1.31	Exercise program	Open	MVPA	90min*3*8weeks	No intervention	13	①③
Benzing et al., 2018 (23)	Switzerland	24/22	10.46 ± 1.35/10.50 ± 1.41	Exergaming	Open	MVPA	15min*1week	Watch video	12	⑥⑦
Kadri et al., 2019 (24)	Tunisia	20/20	14.5 ± 3.5/14.2 ± 3	Taekwondo	Open	MPA	50min*2*72weeks	Routine courses	1	①
Hattabi et al., 2019 (25)	Tunisia	20/20	9.95 ± 1.31/9.75 ± 1.33	Aerobic exercise	Closed	MPA	90min*3*12weeks	No intervention	12	①⑮
Benzing et al., 2019 (26)	Switzerland	28/23	10.46 ± 1.30/10.39 ± 1.44	Exergaming	Open	MVPA	30min*3*8weeks	Wait-list	123	⑥⑦⑧
Chang et al., 2022 (27)	China	16/16	8.36 ± 1.23/8.38 ± 1.31	Table tennis	Open	MPA	60min*3*12weeks	No intervention	13	①⑤
Hattabi et al., 2022 (28)	Tunisia	20/20	9.95 ± 1.31/9.75 ± 1.33	Swim	Closed	MPA	90min*3*12weeks	No intervention	1	⑨
Ludyga et al., 2022 (29)	Switzerland	29/28	10.0 ± 1.2/10.8 ± 1.2	Judo	Open	MPA	60min*2*12weeks	No intervention	3	⑫
Liang et al., 2022 (30)	China	39/39	8.37 ± 1.42/8.29 ± 1.27	Aerobic exercise	Closed	MVPA	60min*3*12weeks	Wait-list	123	④⑥⑭
Chen et al., 2022 (31)	China	32/32	8.37 ± 1.68/7.89 ± 2.13	Aerobic exercise	Closed	MPA	20min*3*12weeks	Watch video	123	①②
Song et al., 2022 (32)	China	8/8	7.68 ± 0.56/7.53 ± 0.79	Football	Open	NR	30min*10*6weeks	No intervention	123	①⑬⑭
Li et al., 2024 (33)	China	16/14	7.93 ± 1.61	Basketball	Open	MVPA	60min*2*8weeks	No intervention	123	①②⑩
Huang et al., 2024 (34)	China	22/16	10.18 ± 1.10/9.15 ± 0.77	Rope skip	Closed	MVPA	60min*2*8weeks	Sport games	2	②
Huang et al., 2025 (35)	China	12/12	10.21 ± 1.27/10.21 ± 1.24	Inline skate	Open	NR	80min*2*12weeks	Wait-list	12	①⑯
Zhu et al., 2026 (36)	China	35/36	8.54 ± 1.34/8.75 ± 1.13	Aerobic exercise	Closed	MVPA	40min*3*12weeks	Educational sessions	123	①⑭⑮
Li et al., 2026 (37)	China	15/13	9.27 ± 1.22/9.23 ± 1.45	Jump rope	Closed	MVPA	60min*2*8weeks	Routine sessions	123	①②⑩
He et al., 2026 错误!未找到引用源。 (38)	China	31/26	7.77 ± 0.88/7.62 ± 0.98	Combined exercise	Open	MVPA	90min*6*3weeks	Wait-list	2	②
Zhang et al., 2026 (39)	China	30/30	8.71 ± 1.26/8.69 ± 1.29	Baduanjin	Closed	MVPA	80min*2*12weeks	Daily routines	1	③

NR, Not Reported; MVPA, Moderate-to-Vigorous Physical Activity; MPA, Moderate Physical Activity; IG, Intervention Group; CG, Control Group.

Executive Function: 1. Inhibition Control; 2. Working Memory; 3. Cognitive Flexibility.

Measures: ① The Stroop Test; ② The N-Back Test; ③ The Go-No-Go Test; ④ The Tower Of London; ⑤ The Wisconsin Card Sorting Test(WCST); ⑥ The Flanker Task; ⑦ The Color Span Backward Task; ⑧ The Simon Task ⑨ The Hayling Test; ⑩ The Task Switch; ⑪ The Brief; ⑫ The Change Detection Paradigm; ⑬ The Rey-Osterrich Complex Figure Test(CFT); ⑭ The Trail Making Test(TMT); ⑮ The Rey Complex Figure Test(ROCF); ⑯ The Spatial Working Memory(SWM); ⑰ The Stop Signal Inhibition Task (STOPIT); ⑱ The Automated Working Memory Assessment System-Short Version (AWMA-S).

### 2.2 Inclusion and exclusion criteria

The inclusion criteria were as follows: RCTs that examined the effect of physical activity on executive function among children with ADHD. To qualify, studied had to meet all of these conditions: (1) participants were children or adolescents aged 6–18 years with a clinical diagnosis of ADHD; (2) the intervention involved structured physical activity, including exercise, sports, or organized physical training; (3) the control group received wait-list care or alternative treatments, such as art project, routine courses or watching video; (4) outcomes measured included executive function or at least one of its three core subcomponents (e.g., inhibitory control, working memory, and cognitive flexibility); (5) the studies were published as randomized controlled trials (RCTs) with no statistically significant between-group differences at baseline. In addition, requirements included complete reporting of pre- and post-intervention data (means, standard deviations, and sample sizes) and publication in either English or Chinese.

The exclusion criteria were as follows: (1) non-RCTs, duplicate publications, reviews, conference abstracts, animal experiments, or case reports; (2) articles lacking essential statistical data (e.g., those reporting only pre- or post-intervention values); (3) irrelevant topics, inaccessible full texts, or publications in languages other than English or Chinese.

### 2.3 Data extraction

Both the screening and data extraction processes were conducted independently by two researchers (Chen CZ and Fan MY). Any discrepancies were resolved through consultation with a third reviewer, who jointly made the final decision. Information collected from each study included: first author, publication year, country, sample size, mean age, and gender distribution of participants. Detailed intervention characteristics were also extracted, including the type, frequency, duration, and intensity of physical activity, as well as the nature of the control condition. In addition, data on outcome measures were recorded, including the specific instruments used to assess executive function and its subcomponents. This procedure ensured the accuracy and consistency of the extracted data.

### 2.4 Quality assessment

The risk of bias for the included studies was assessed using the Cochrane Risk of Bias 2.0 (RoB 2.0) tool. RoB 2.0 evaluates bias across five core domains: “randomisation process” “deviations from the intended interventions” “missing outcome data” “measurement of the outcome” and “selection of the reported result”. The overall risk of bias is categorized as “low risk of bias” “some concerns” or “high risk of bias” (40). The assessment of the risk of bias was performed by two evaluators independently and verified each other (Chen CZ and Fan MY), and in case of disagreement, a third evaluator made the decision.

### 2.5 Statistical analysis

All statistical analyses were performed using The Review Manager version 5.4 (RevMan 5.4). For continuous outcomes, standardized mean differences (SMDs) with 95% confidence intervals (CIs) were calculated to estimate pooled effect sizes. For effect size coding, negative SMD values were predefined to indicate a beneficial effect of the intervention compared with the control group. To ensure consistent interpretation, outcome measures where higher scores represented worse performance (e.g., reaction time, error rate) were reverse-coded before analysis, such that all values were oriented in the same direction: lower scores on the original scales were recoded to reflect better performance, resulting in negative SMDs when the intervention group outperformed the control group.

A two-tailed P value of <0.05 was considered statistically significant. Heterogeneity across studies was assessed using the I^2^ statistic. When I^2^ was <50%, a fixed-effects model was applied, indicating low heterogeneity. For moderate heterogeneity (50% ≤ I^2^ < 75%), a random-effects model was used. In cases of substantial heterogeneity (I^2^ ≥ 75%), subgroup analyses were conducted to explore potential sources of variability. Sensitivity analyses were performed by switching between fixed- and random-effects models and by sequentially excluding individual studies to examine the robustness of the pooled results in terms of SMD, 95% CI, and I^2^. Effect sizes with absolute SMD values of approximately 0.2, 0.5, and 0.8 were interpreted as small, moderate, and large, respectively, based on conventional benchmarks. Egger’s test with STATA 19.0 software was used to test publication bias. When p > 0.05, no bias exists; when p ≤ 0.05, publication bias may exist.

## 3 Results

### 3.1 Literature search and screening

**Figure 1:**
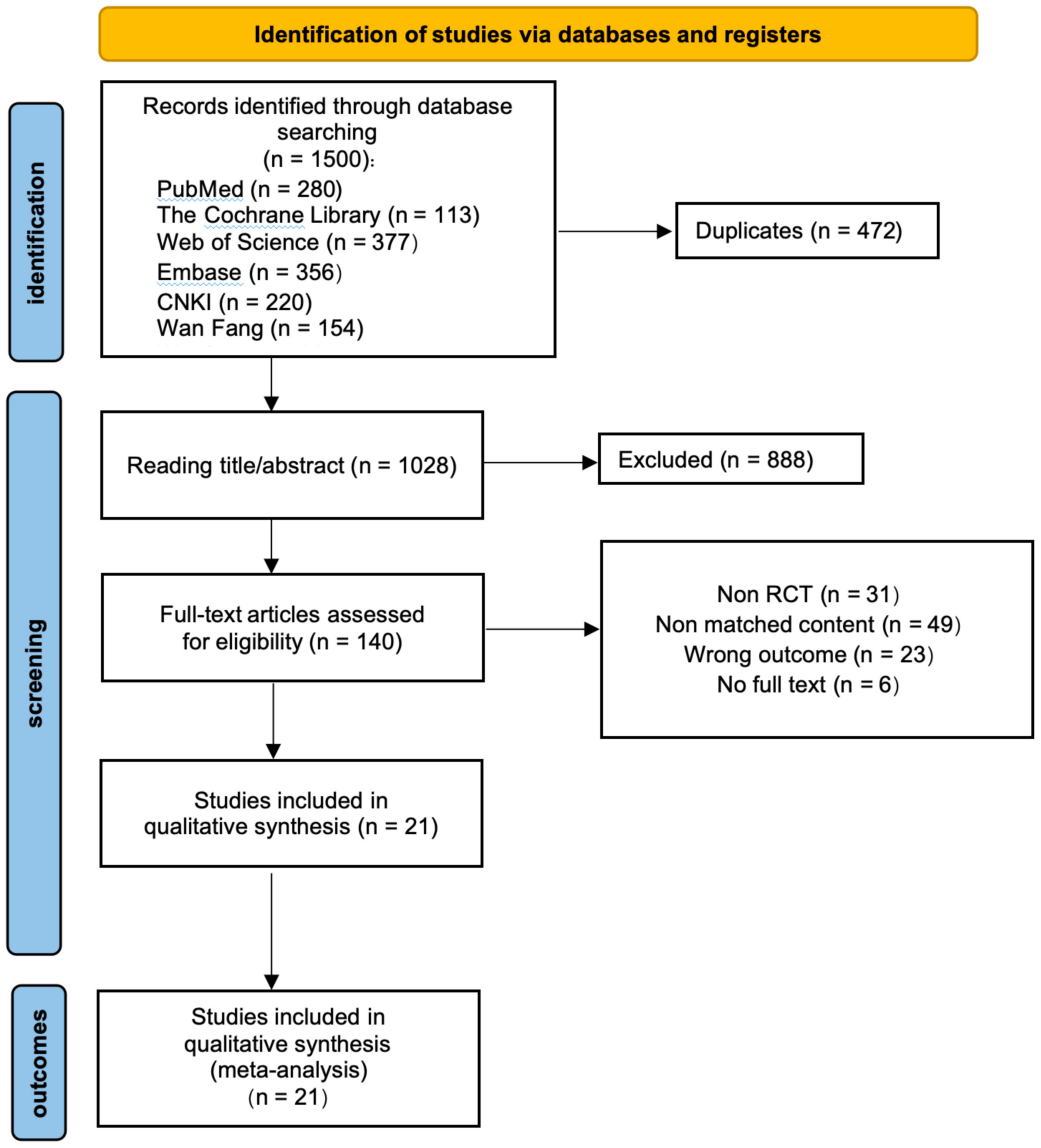
The PRISMA flow diagram of study selection.

A total of 1,500 records were initially identified through six electronic databases: PubMed (n = 280), The Cochrane Library (n = 113), Web of Science (n = 377), Embase (n = 356), CNKI (n = 220), and Wanfang Data (n = 154). After importing all records into EndNote 21 and removing 472 duplicates, 1,028 unique articles remained for screening. Based on titles and abstracts, 888 records were excluded because they did not meet the eligibility criteria. During the full-text assessment, 31 studies were excluded because they did not employ a randomized controlled trial design, 49 were deemed irrelevant, 23 lacked outcome measures related to executive function, and 6 were inaccessible due to the unavailability of the full text. After applying all prespecified inclusion and exclusion criteria, 21 studies were finally included in this meta-analysis. The complete study selection process is summarized in **Figure 1**, following the PRISMA flow diagram format.

### 3.2 Basic characteristics of included studies

All 21 studies included in this meta-analysis were RCTs. In the intervention groups, various forms of structured physical activity were implemented, whereas control groups engaged in standard physical education classes, routine physical activities, sedentary behaviors, or video-watching conditions. The primary outcomes focused on the three core components of executive function—inhibitory control, working memory, and cognitive flexibility—assessed using well-established and validated instruments commonly applied in executive function research. The included studies were conducted across several countries, including the United States, China, Tunisia, Iran, and Switzerland. Participants’ mean ages ranged from 7 to 14 years. Two studies adopted a short-term design (19, 23), consisting of a single intervention session delivered within one week, whereas the remaining studies applied intervention durations ranging from 3 to 12 weeks, and only one study had a relatively long intervention duration, with a period of about 72 weeks (24), typically with at least two sessions per week. Session duration varied across studies: Seven investigations employed sessions lasting 15–50 minutes (19, 23, 24, 26, 31, 32, 36), while Fourteen studies implemented sessions of 60 minutes or longer (20–22, 25, 27–30, 33–35, 37) 错误!未找到引用源。 (39). Seven studies adopted moderate intensity (19, 24, 25, 27–29, 31), three studies did not report intensity information (21, 32, 35), and eleven studies employed moderate-to-vigorous intensity (20, 22, 23, 26, 30, 33, 34, 36, 37) 错误!未找到引用源。 (39).

All interventions were classified as aerobic physical activities, and for analytical purposes, they were further categorized into three main comparative dimensions. First, regarding activity openness, nine studies examined closed activities (19, 25, 28, 30, 32, 34, 36, 37, 39), while twelve studies investigated open activities (20–24, 26, 27, 29, 31, 33, 35) 错误!未找到引用源。. Second, concerning intervention composition, seven studies implemented multi-component physical activity programs (20, 22, 23, 25, 26, 30) 错误!未找到引用源。, whereas fourteen studies involved single aerobic activities (21, 24, 27–29, 31–37, 39). Third, in terms of activity type, three studies focused on traditional aerobic activities such as running and cycling (19, 31, 36), four studies examined ball sports (21, 27, 32, 33), two studies investigated aquatic activities (25, 28), six studies involved high-cognitive-engagement physical activities (20, 22, 23, 26, 30) 错误!未找到引用源。 and six studies involved leisure-fitness activities (24, 29, 34, 35, 37, 39). Control groups across the included studies participated in standard physical education classes such as art project and sport games, waiting-list control conditions, or sedentary activities such as video watching. A detailed summary of the characteristics of the included studies is presented in **Table 1**.

### 3.3 Quality assessment of included studies

**Figure 2:**
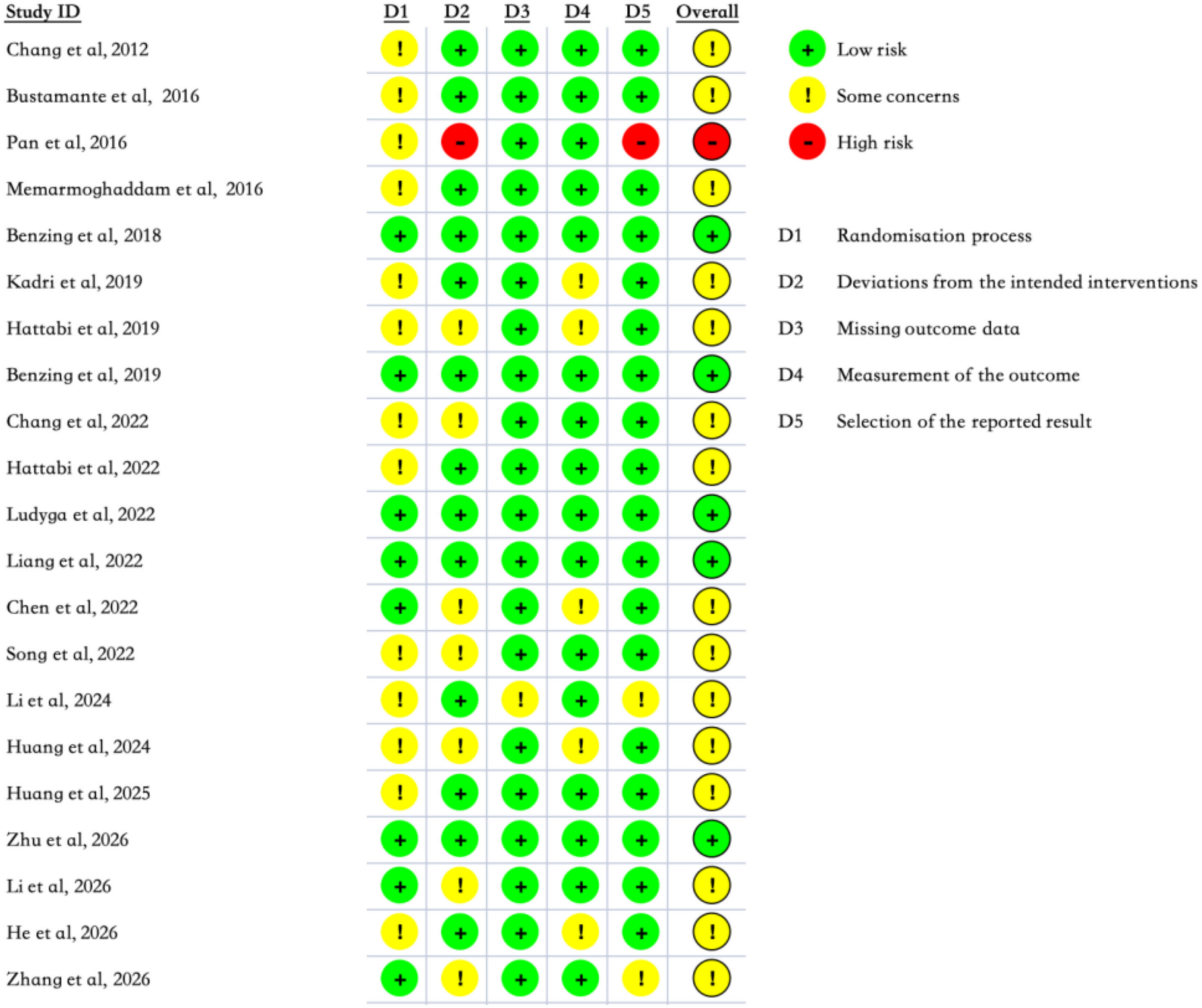
Summary of the risk of bias assessment.

The methodological quality of the included studies was assessed in accordance with the Cochrane Risk of Bias 2.0 (RoB 2.0) tool. 24% of the studies were rated at low risk, 71% of the studies were rated at some concerns and only 5% of the studies were rated at high risk. The summary of the risk of bias assessment are shown in **Figure 2**.

### 3.4 Meta-analysis results

#### 3.4.1 Overall effects of physical activity on inhibitory control

A total of 18 studies reporting inhibitory control outcomes were included, involving 763 participants (389 in the intervention group and 374 in the control group). The pooled analysis indicated significant heterogeneity among studies (I^2^ = 38%, P = 0.05). Using a fix-effects model, physical activity showed a significant beneficial effect on inhibitory control in children with ADHD (SMD = -0.69, 95% CI [-0.84, -0.54], P<0.00001).

**Figure 3:**
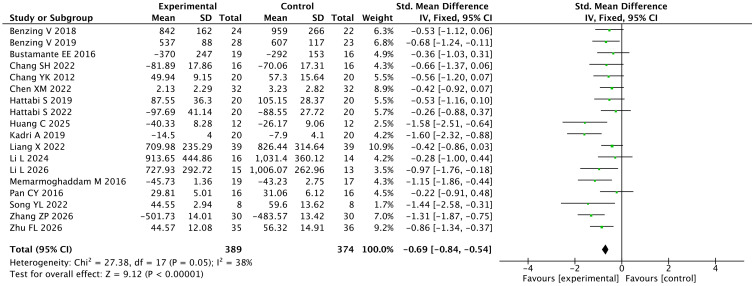
Forest plot of physical activity effects on inhibitory control in ADHD children.

Sensitivity analyses were performed by sequentially removing individual studies, yielding SMD values ranging from -0.64 to -0.72 and I^2^ values ranging from 24% to 42%, with no change in the direction of the pooled effect. These results indicate that the findings are robust and not overly influenced by any single study. The forest plot summarizing these results is shown in **Figure 3**.

Subgroup analyses were performed to identify potential sources of variability. Subgroups were stratified according to intervention intensity, intervention duration (weeks), single-session duration and frequency. The results in **Table 2** indicated that both MPA (6 of the studies) and MVPA (9 of the studies) physical activities produced a statistically significant improvement in inhibitory control (P<0.00001). For intervention duration, both 2 studies lasting less than 6 weeks and 16 studies lasting 6 or more weeks showed significant improvement. However, the longer interventions lasting 6 or more weeks (SMD=-0.71, P < 0.00001) showed larger effect sizes than less than 6 weeks (SMD=-0.54, P = 0.01). Regarding single-session duration, 7 studies lasting 15–50 minutes demonstrated superior effects (SMD=-0.75, P<0.00001) compared with 11 studies lasting 60–90 minutes (SMD=-0.65, P<0.00001). For intervention frequency, all groups showed significant improvement. 6 twice-weekly studies showed the largest effect size (SMD=-1.01, P<0.0001) compared with 3 once-weekly studies (SMD=-0.49, P = 0.008) and 9 studies lasting 3 or more times one week (SMD=-0.60, P<0.0001).

**Table 2:** Result of subgroup analysis on inhibitory control.

Parameters	Type	Number	SMD	95%CI	Z	*I^2^*	*P*
Intensity	MPA	6	-0.61	-0.86, -0.36	4.71	45%	< 0.00001
	MVPA	9	-0.71	-0.91, -0.52	7.14	27%	< 0.00001
Weeks	< 6weeks	2	-0.54	-0.98, -0.11	2.47	0%	0.01
	≥ 6weeks	16	-0.71	-0.87, -0.55	8.81	44%	< 0.00001
Times	15-50min	7	-0.75	-0.97, -0.52	6.46	37%	< 0.00001
	60-90min	11	-0.65	-0.84, -0.45	6.48	43%	< 0.00001
Frequency	1/week	3	-0.49	-0.85, -0.13	2.65	0%	0.008
	2/week	6	-1.01	-1.49, -0.52	4.05	64%	< 0.0001
	≥ 3/week	9	-0.60	-0.80, -0.40	5.95	0%	< 0.00001

These findings suggest that moderate-to-vigorous intensity activity, intervention periods of at least 6 weeks, session durations of 15–50 minutes and twice-weekly physical activities are optimal parameters for enhancing inhibitory control in children with ADHD.

We grouped the included studies into three categories: (1) comparison between open and closed physical activities, (2) comparison between single aerobic activities and multi-component combination activities, and (3) comparison among aquatic activities, ball activities, traditional aerobic activities (running and cycling), high-cognitive-engagement activities and leisure-fitness activities. The results in **Table 3** indicated that 10 studies of open activities were more effective than 8 studies of closed activities in enhancing inhibitory control. 12 studies of single aerobic activities and 6 studies of multi-component combination activities helped improve inhibitory control. Single aerobic activities (SMD=-0.79, P<0.00001) had better improvement than multi-component activities (SMD=-0.57, P<0.00001). 4 studies of leisure-fitness activities (SMD=-1.35, P<0.0001) showed a strong effect in boosting inhibitory control in children with ADHD compared to 2 studies of aquatic activities, 3 studies of traditional aerobic activities, 4 studies of ball activities and 5 studies of high-cognitive-engagement activities.

**Table 3:** Results of analysis of different forms of physical activity on inhibitory control.

Type	Number	SMD	95%CI	Z	*I^2^*	*P*
OPEN	10	-0.75	-0.97, -0.52	6.56	46%	< 0.00001
CLOSED	8	-0.64	-0.84, -0.45	6.38	31%	< 0.00001
single aerobic activities	12	-0.79	-1.07, -0.51	5.60	51%	< 0.00001
multi-component activities	6	-0.57	-0.81, -0.33	4.70	0%	< 0.00001
aquatic activities	2	-0.50	-0.89, -0.12	1.73	21%	0.08
ball activities	4	-0.97	-1.80, -0.15	2.57	85%	0.02
traditional aerobic activities	3	-0.62	-0.93, -0.32	4.02	0%	< 0.0001
high-cognitive-engagement	5	-0.58	-0.83, -0.32	4.40	0%	< 0.0001
leisure-fitness activities	4	-1.35	-1.70, -0.99	7.39	0%	< 0.00001

#### 3.4.2 Overall effects of physical activity on cognitive flexibility

11 studies reporting outcomes related to cognitive flexibility were included, involving a total of 503 participants (257 in the intervention group and 246 in the control group). The pooled analysis showed moderate heterogeneity among studies (I^2^ = 0%, P = 0.88). Using a fix-effects model, physical activity was found to have a significant positive effect on cognitive flexibility in children with ADHD (SMD = -0.53, 95% CI [-0.71, -0.35], P < 0.00001).

**Figure 4:**
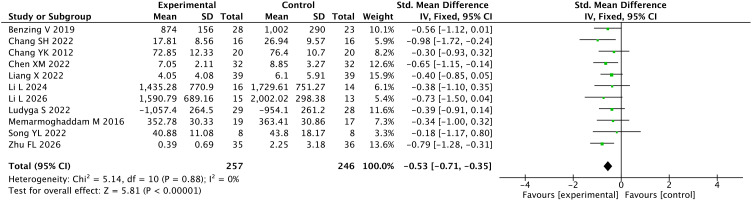
Forest plot of physical activity effects on cognitive flexibility in ADHD children.

Sensitivity analyses were conducted by sequentially excluding individual studies. The resulting SMD values ranged from -0.49 to -0.55, and I^2^ values ranged from 0% to 0%, with no change in the direction of the pooled effect and all results remaining statistically significant (P < 0.05). These findings indicate that the beneficial effect of physical activity on cognitive flexibility is robust and not driven by any single study. The forest plot summarizing these results is presented in **Figure 4**.

Subgroup analyses were conducted to explore potential sources of variability. Subgroups were defined according to intervention intensity, intervention duration (weeks), and single-session duration and frequency. The results in **Table 4** showed that both 4 studies of MPA activities (P = 0.0002) and 6 studies of MVPA activities (P<0.00001) produced significant improvements in cognitive flexibility. In terms of intervention duration, 10 studies lasting at least 6 weeks yielded significant benefits (P<0.00001), whereas 1 study lasting less than 6 weeks was associated with weaker effects (P = 0.34). Regarding single-session duration, 5 studies lasting 15–50 minutes showed a stronger effect (SMD= -0.58, P< 0.0001) compared with 6 studies lasting 60–90 minutes (SMD= -0.49, P = 0.0001). For intervention frequency, 7 studies lasting 3 or more times one week showed the largest effect size (SMD=-0.58, P<0.0001) compared with 1 once-weekly studies (SMD=-0.30, P = 0.34) and 3 twice-weekly studies (SMD=-0.46, P = 0.01).

**Table 4:** Result of subgroup analysis on cognitive flexibility.

Parameters	Type	Number	SMD	95%CI	Z	*I^2^*	*P*
Intensity	MPA	4	-0.54	-0.83, -0.26	3.70	0%	0.0002
	MVPA	6	-0.54	-0.77, -0.31	4.52	0%	< 0.00001
Weeks	< 6weeks	1	-0.30	-0.93, 0.32	0.95	0	0.34
	≥ 6weeks	10	-0.55	-0.74, -0.36	5.78	0%	< 0.00001
Times	15-50min	5	-0.58	-0.84, -0.32	4.39	0%	< 0.0001
	60-90min	6	-0.49	-0.73, -0.24	3.84	0%	0.0001
frequency	1/week	1	-0.30	-0.93, 0.32	0.95	0	0.34
	2/week	3	-0.46	-0.84, -0.09	2.45	0%	0.01
	≥ 3/week	7	-0.58	-0.79, -0.36	5.26	0%	< 0.00001

We grouped the included studies into three categories: (1) comparison between open and closed physical activities, (2) comparison between single aerobic activities and multi-component activities, and (3) comparison among aquatic activities, ball activities, traditional aerobic activities (running and cycling), high-cognitive-engagement activities and leisure-fitness activities. The results in **Table 5** showed that 6 studies of closed physical activities (SMD=-0.61, P<0.00001) had a moderate effect in improving cognitive flexibility than 5 studies of open physical activities (SMD=-0.40, P = 0.006). Both 8 studies of single aerobic exercise (SMD=-0.58, P<0.00001) and 3 studies of multi-component exercises (SMD=-0.43, P = 0.006) were effective in improving cognitive flexibility in children with ADHD. 3 studies of traditional aerobic activities showed a better improvement in improving cognitive flexibility (SMD=-0.62, P<0.0001) than aquatic activities, 3 studies of ball activities (SMD=-0.57, P = 0.02), 3 studies of high-cognitive-engagement activities (SMD=-0.43, P = 0.006) and 2 studies of leisure-fitness activities (SMD=-0.50, P = 0.02).

**Table 5:** Results of analysis of different forms of physical activity on cognitive flexibility.

Type	Number	SMD	95%CI	Z	*I^2^*	*P*
OPEN	5	-0.40	-0.69, -0.12	2.74	0%	0.006
CLOSED	6	-0.61	-0.84, -0.38	5.24	0%	< 0.00001
single aerobic activities	8	-0.58	-0.80, -0.36	5.17	0%	< 0.00001
multi-component activities	3	-0.43	-0.74, -0.12	2.75	0%	0.006
aquatic activities	0	0	0	0	0	0
ball activities	3	-0.57	-1.02, -0.11	2.42	3%	0.02
traditional aerobic activities	3	-0.62	-0.93, -0.32	4.01	0%	< 0.0001
high-cognitive-engagement	3	-0.43	-0.74, -0.12	2.75	0%	0.006
leisure-fitness activities	2	-0.50	-0.93, -0.06	2.25	0%	0.02

#### 3.4.3 Overall effects of physical activity on working memory

**Figure 5:**
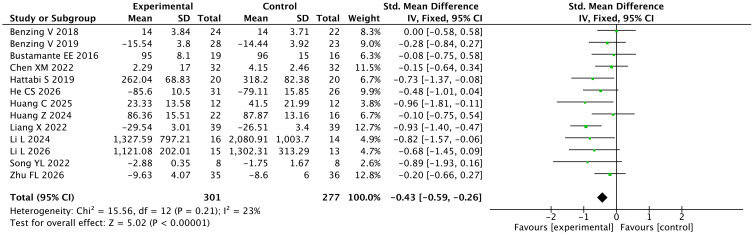
Forest plot of physical activity effects on working memory in ADHD children.

13 studies reporting working memory outcomes were included, involving a total of 578 participants (301 in the intervention group and 277 in the control group). The pooled analysis showed relatively low heterogeneity (I^2^ = 23%, P = 0.21); therefore, a fixed-effects model was used. Physical activity had a significant positive effect on working memory in children with ADHD (SMD = -0.43, 95% CI [-0.59, −0.26], P < 0.00001). Sensitivity analyses were performed by sequentially removing individual studies. The resulting SMD values ranged from -0.35 to -0.46 and I^2^ values ranged from 0% to 29%, with no change in the overall effect direction and all results remaining statistically significant (P < 0.05). These findings suggest that the beneficial impact of physical activity on working memory is robust and not overly influenced by any single study. The forest plot illustrating these results is shown in **Figure 5**.

Despite the low heterogeneity, a subgroup analysis was still conducted for the working memory group to better identify the sources of heterogeneity. The results in **Table 6** showed that 11 studies of MVPA activities produced significant improvements in working memory (SMD=-0.44, P<0.00001), whereas 2 studies of MPA activities showed no significant improvement (SMD=-0.36, P = 0.07). 11 studies lasting at least 6 weeks produced significant improvements in working memory (SMD=-0.44, P<0.00001), whereas 2 studies lasting less than 6 weeks showed no significant improvement (SMD=-0.39, P = 0.05). 8 studies lasting 60-90min (SMD=-0.60, P<0.00001) had a moderate effect in improving working memory in children with ADHD than 5 studies lasting 15-50min (SMD=-0.21, P = 0.11). 2 once-weekly activities showed no significant improvement in improving working memory (SMD=-0.04, P = 0.87). 5 twice-weekly studies (SMD=-0.57, P = 0.002) showed a better improvement in working memory than 7 studies lasting less than 3 times a week (SMD=-0.47, P<0.00001).

**Table 6:** Result of subgroup analysis on working memory.

Parameters	Type	Number	SMD	95%CI	Z	*I^2^*	*P*
Intensity	MPA	2	-0.36	-0.75, 0.03	1.82	48%	0.07
	MVPA	11	-0.44	-0.63, -0.26	4.69	26%	< 0.00001
Weeks	< 6weeks	2	-0.39	-0.77, -0.00	1.98	0%	0.05
	≥ 6weeks	11	-0.44	-0.62, -0.25	4.62	34%	< 0.00001
Times	15-50min	5	-0.21	-0.46, 0.05	1.61	0%	0.11
	60-90min	8	-0.60	-0.83, -0.38	5.29	12%	< 0.00001
frequency	1/week	2	-0.04	-0.47, 0.40	0.16	0%	0.87
	2/week	4	-0.57	-0.94, -0.20	3.03	10%	0.002
	≥ 3/week	7	-0.47	-0.68, -0.26	4.46	28%	< 0.00001

We grouped the included studies into three categories: (1) comparison between open and closed physical activities, (2) comparison between single aerobic activities and multi-component activities, and (3) comparison among aquatic activities, ball activities, traditional aerobic activities (running and cycling), high-cognitive-engagement activities and leisure-fitness activities. The results in **Table 7** showed that both open and closed physical activities improved working memory in children with ADHD, with 6 studies of closed physical activities (SMD=-0.45, P<0.0001) showing a more significant improvement than 7 studies of open sports activities (SMD=-0.40, P = 0.002). 6 studies of multi-component activities (SMD=-0.46, P<0.0001) had a better improvement than 7 studies of single aerobic activities (SMD=-0.39, P = 0.002). 2 studies of traditional aerobic activities (P = 0.31) had no moderate effect in working memory in children with ADHD. 2 studies of ball activities (SMD=-0.84, P = 0.007) had a better improvement in working memory than 5 studies of high-cognitive-engagement (SMD=-0.43, P = 0.0007), 3 studies of leisure-fitness activities (SMD=-0.50, P = 0.02) and 1 study of aquatic activity (SMD=-0.73, P = 0.03).

**Table 7:** Results of analysis of different forms of physical activity on working memory.

Type	Number	SMD	95%CI	Z	*I^2^*	*P*
OPEN	7	-0.40	-0.65, -0.15	3.13	10%	0.002
CLOSED	6	-0.45	-0.68, -0.23	3.94	43%	< 0.0001
single aerobic activities	7	-0.39	-0.63, -0.14	3.10	10%	0.002
multi-component activities	6	-0.46	-0.69, -0.24	3.97	42%	< 0.0001
aquatic activities	1	-0.73	-1.37, -0.08	2.21	0	0.03
ball activities	2	-0.84	-1.45, -0.23	2.70	0%	0.007
traditional aerobic activities	2	-0.18	-0.51, 0.16	1.02	0%	0.31
high-cognitive-engagement	5	-0.43	-0.67, -0.18	3.41	50%	0.0007
leisure-fitness activities	3	-0.50	-0.92, -0.07	2.27	28%	0.02

#### 3.4.4 Publication bias

**Figure 6:**
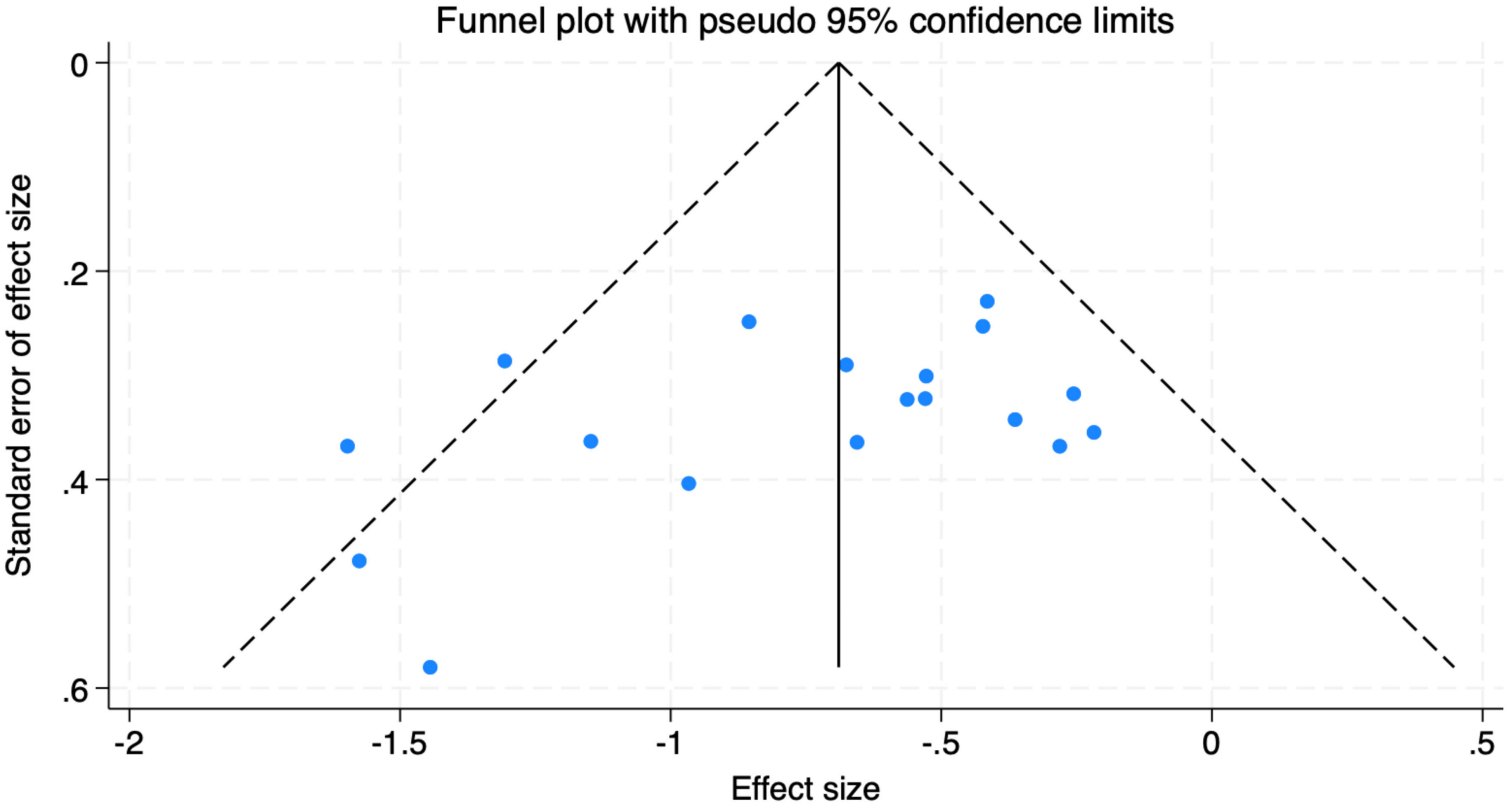
Funnel plot assessing publication bias for inhibitory control under the reverse scoring.

**Figure 7:**
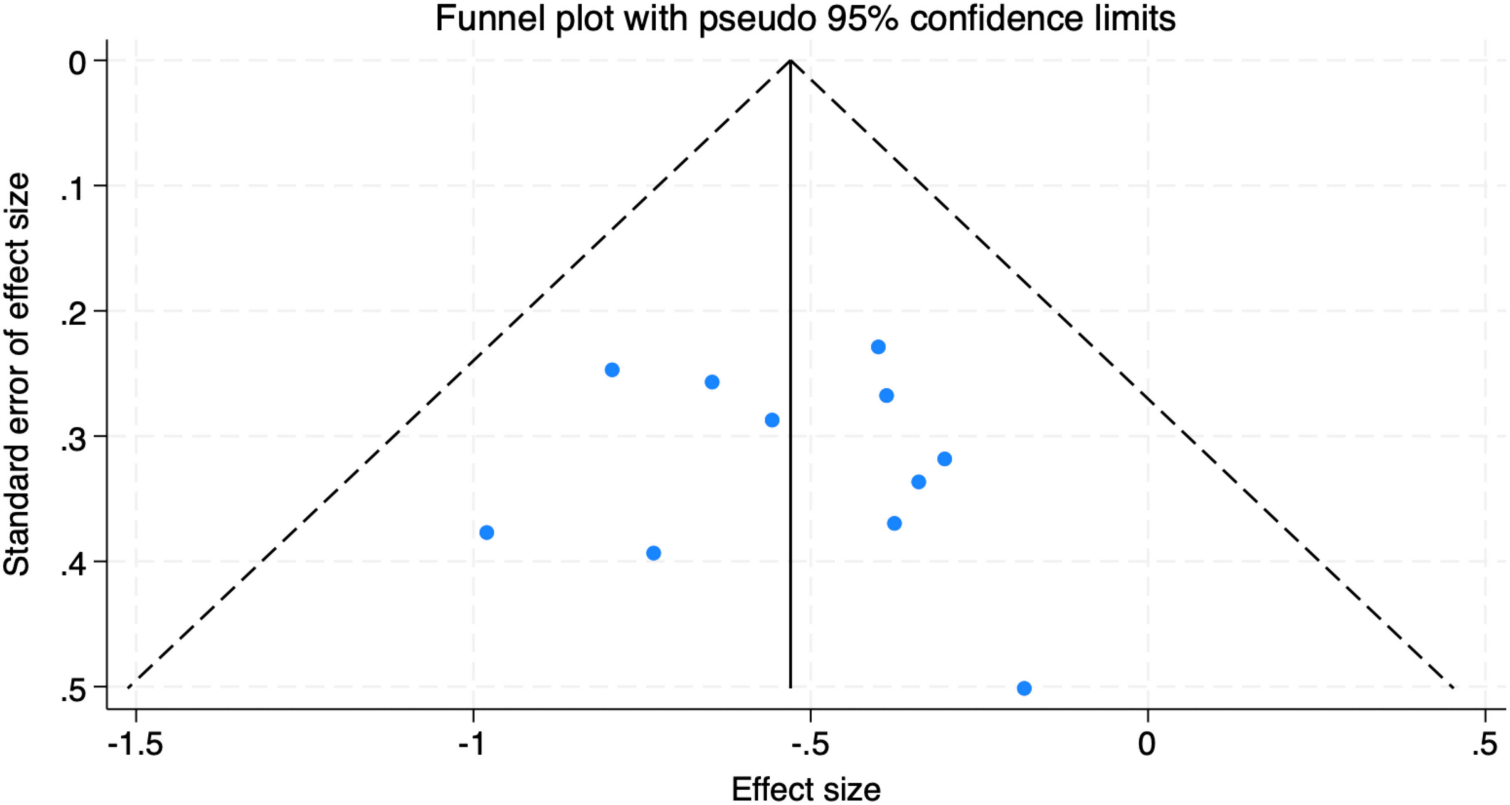
Funnel plot assessing publication bias for cognitive flexibility under the reverse scoring.

**Figure 8:**
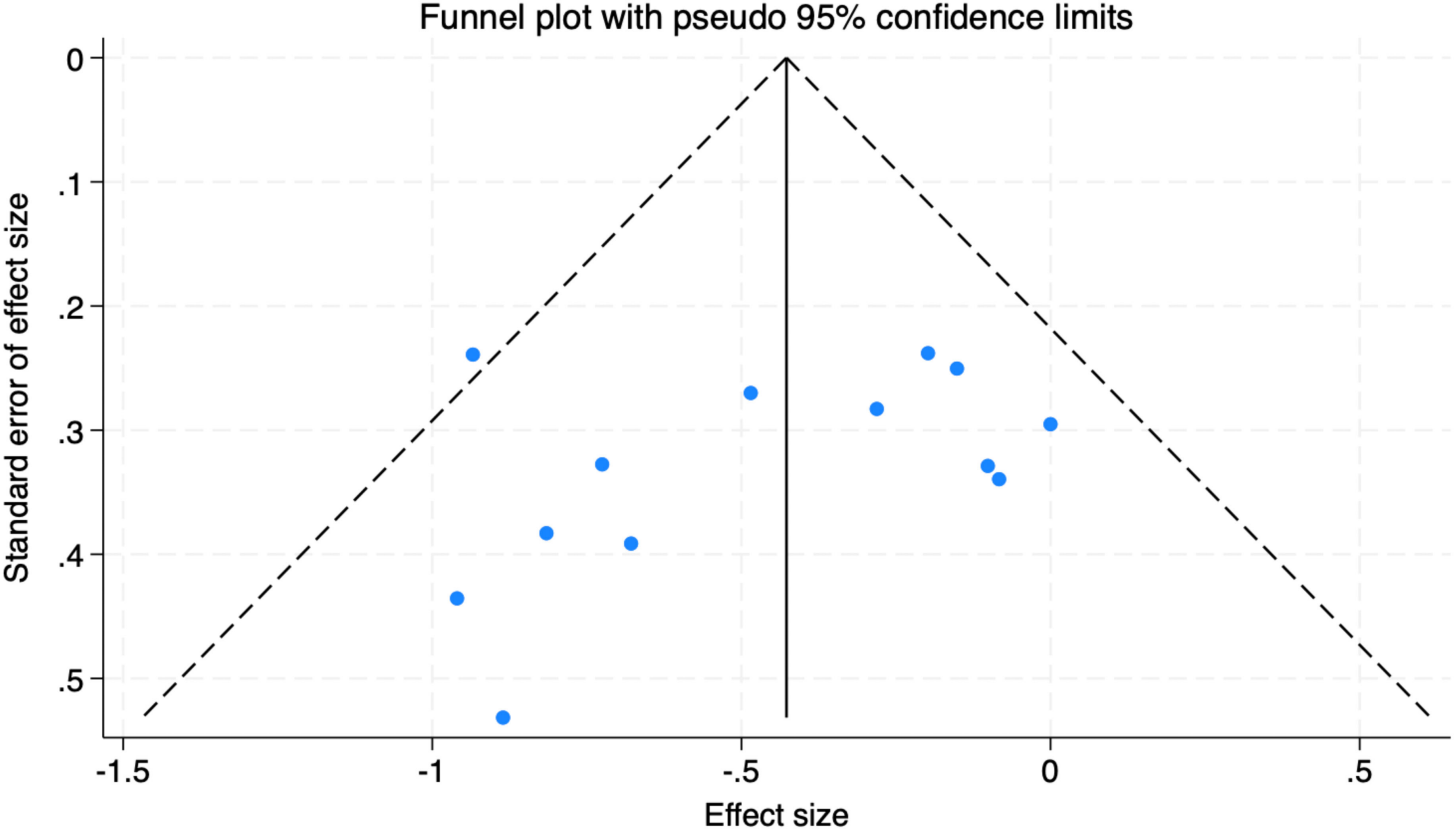
Funnel plot assessing publication bias for working memory under the reverse scoring.

The results of Egger’s tests indicated that the p values of inhibitory control, cognitive flexibility and working memory were 0.103, 0.769 and 0.299 (**Table 8**). The results suggested no remarkable publication bias. The funnel plots showed the same result (**Figures 6**–**8**).

**Table 8:** Egger’s outcome for publication bias.

Outcomes	N	t	Egger’s outcomes	Publication bias
inhibitory control	18	-1.73	0.103	No
cognitive flexibility	11	0.30	0.769	No
working memory	13	-1.09	0.299	No

## 4 Discussion

### 4.1 Main findings

The present meta-analysis demonstrated that physical activity significantly enhanced all three core components of executive function in children with ADHD, including inhibitory control (SMD = −0.69, 95% CI [−0.84, −0.54], P<0.00001), cognitive flexibility (SMD = −0.53, 95% CI [−0.71, −0.35], P<0.00001), and working memory (SMD = −0.43, 95% CI [−0.59, −0.26], P<0.00001). These findings indicate that engaging in structured physical activity exerts a robust and broadly positive effect on executive function in children with ADHD, which is consistent with previous reports in this field (41).

Several primary studies included in our review support these conclusions. For example, one randomized trial reported that a single five-minute bout of high-intensity activity significantly improved executive function in children with ADHD, suggesting that even very brief exercise sessions can elicit immediate cognitive benefits (42). Treadmill-based interventions delivered over eight weeks have also been shown to ameliorate executive function deficits in this population (43). In addition, a recent meta-analysis of non-pharmacological interventions confirmed that physical exercise exerts substantial effects on specific executive function domains, particularly inhibitory control and cognitive flexibility (44).

From a neurobiological perspective, executive function is closely linked to the maturation and functional integrity of the prefrontal cortex and basal ganglia Executive function is closely associated with the maturation of the prefrontal cortex and basal ganglia (45), and ADHD has been associated with dysregulation of dopaminergic and noradrenergic neurotransmission (46). Physical activity can increase the release of dopamine and norepinephrine, thereby modulating neural circuits involved in inhibitory control in children with ADHD (47). Chan et al. (48) further demonstrated that aerobic exercise promotes the secretion of neurotransmitters such as serotonin and dopamine and activates central nervous system pathways. Medina et al. (49) reported that physical activity of sufficient intensity can upregulate brain-derived neurotrophic factor (BDNF), enhancing neuroplasticity. Long-term participation in physical activity may substantially elevate BDNF levels, thereby improving neuroplasticity in the prefrontal cortex and hippocampus (3). These adaptations can accelerate information processing and improve task execution, enabling children with ADHD to perform better on complex cognitive tasks (50) and ultimately to achieve greater gains in executive function. In light of these findings, encouraging children with ADHD to participate regularly in appropriately designed physical activity programs, rather than maintaining predominantly sedentary lifestyles, may represent a practical and biologically plausible approach to improving executive function in this population.

### 4.2 Subgroup analysis: effects of different physical activity characteristics on executive function

This study took the cycle, duration and intensity of physical activity as intervention variables, and conducted a subgroup analysis on the three core subcomponents of executive function one by one. In terms of intervention intensity, MVPA yielded superior improvements in executive function among children with ADHD. This finding is inconsistent with a previous study which concluded that compared with MVPA, MPA produces a moderate-to-large training effect on overall executive function (51). The present study found no significant effect of MPA on improving working memory in children with ADHD. Some scholars have argued that MPA represents the most effective dose for improving cognition and brain health (52). Other studies have indicated that moderate-to-vigorous physical activity is more strongly associated with cognitive improvement (52). Additionally, MVPA intervention is more suitable for enhancing cognitive flexibility in children with ADHD, which deviates from the findings of prior studies (9, 34).

In terms of intervention duration, cycle and frequency, physical activity lasting at least 6 weeks, twice a week for 15–50 minutes per session produced the optimal improvement in inhibitory control; interventions lasting at least 6 weeks, three times a week for 15–50 minutes per session achieved the best effect on cognitive flexibility; and interventions lasting at least 6 weeks, twice a week for 60–90 minutes per session yielded the greatest improvement in working memory. Accordingly, long-term physical activity significantly improves executive function in children with ADHD, which is supported by previous evidence (53, 54). By contrast, short-term acute physical activity shows no obvious beneficial effect on executive function relative to long-term intervention, which is inconsistent with earlier research (55–57). Hung et al. (55) reported that a single bout of acute moderate-intensity aerobic exercise exerts a positive influence on working memory in children with ADHD. Pontifex et al. (56) demonstrated that one-session moderate-intensity aerobic exercise can improve inhibitory control among ADHD children. A meta-analysis by Chen et al. (57) involving 13 studies suggested that a single session of physical activity only yields a small improvement in executive function. The inconsistency may stem from differences in literature inclusion criteria: the present study incorporated diverse forms of physical activity rather than a single type of aerobic exercise. Furthermore, age heterogeneity and varying degrees of executive function impairment among participants may also account for the divergent results. Compared with single-session physical activity, long-term physical activity interventions are more effective in improving executive function in children with ADHD, and improvement in working memory requires longer-term physical activity participation. Therefore, from a long-term perspective, single and short-term physical activity interventions are not recommended for children with ADHD. Instead, long-term interventions lasting at least 6 weeks with no fewer than two sessions per week are highly recommended to optimize executive function outcomes.

Analysis of different physical activity forms revealed that open physical activities exert a relatively better effect on improving inhibitory control than closed physical activities, while closed activities are superior in enhancing cognitive flexibility and working memory in children with ADHD. Open physical activities are performed in dynamic and unpredictable environments, requiring participants to respond rapidly to changing situations rather than following fixed patterns. Such activities demand multi-information processing and rapid cognitive switching under diverse tasks, strategies and rules. In contrast, closed physical activities are conducted in relatively stable and predictable settings with little external interference and lower cognitive resource consumption, where all movements and skills are performed within a controllable scope. Hence, open-skill physical activities place higher demands on inhibitory control, requiring advanced cognitive processing and task switching as well as greater brain involvement in information processing, which aligns with the viewpoints of previous systematic reviews (58). Meanwhile, Diamond et al. (59) pointed out that open activities such as martial arts and team sports significantly enhance executive function in children and adolescents due to their high cognitive demands and dynamic environmental characteristics. In terms of working memory and cognitive flexibility, closed physical activities show better therapeutic effects than open ones. A plausible explanation is that closed-skill activities enable participants to exercise at a self-regulated pace in a controllable and stable environment with low cognitive load, facilitating motor skill acquisition and cardiorespiratory fitness improvement. Notably, enhanced cardiorespiratory fitness is closely correlated with neuroelectrical indicators of working memory (60). The discrepancies may also be attributed to limited sample sizes and inconsistent study populations across included literature.

Regarding single aerobic physical activity and multi-component physical activity interventions, the results indicated that both approaches can effectively improve executive function in children with ADHD. Specifically, single aerobic physical activity is superior in improving inhibitory control and cognitive flexibility, whereas multi-component physical activity yields better effects on working memory (61). The difference may be explained by the fact that multi-component interventions contain diverse and complex activity components that require stronger information processing and frequent task switching. In addition, all included studies were dominated by aerobic exercise, and multi-component interventions were mainly composed of aerobic exercise combined with other physical fitness or neurocognitive tasks. This study holds that multi-component physical activities incorporating aerobic exercise have a favorable effect on executive function improvement in children with ADHD, which has been verified by previous scholarly research (62). Therefore, it is recommended that children with ADHD engage in more challenging multi-component physical activities integrated with aerobic exercise.

The results also showed that leisure-fitness physical activities are optimal for improving inhibitory control; traditional aerobic activities such as running and cycling are more beneficial for cognitive flexibility; and ball sports achieve the best improvement in working memory among children with ADHD, which is consistent with previous studies (33, 41). Physical activities with high cognitive involvement were found to improve all three dimensions of executive function, possibly because such activities require greater sustained attention and cognitive engagement. Meta-analysis have confirmed that cognitively challenging physical activities are more effective in promoting cognitive ability than simple aerobic exercise (63, 64), which supports the conclusions of the present study. Physical activities with higher cognitive demands demonstrate greater advantages in improving cognitive flexibility than other activity types. No significant effect of aquatic physical activity on inhibitory control or of traditional aerobic activity on working memory was observed in this study. The null findings may be due to the limited number of eligible studies, making it impossible to determine the exact effects of aquatic activities on these two indicators. Future research should incorporate more qualified literature to further explore this issue.

### 4.3 Limitations

This study has several limitations that should be considered when interpreting the findings. First, substantial variation existed in the types of physical activity interventions, assessment tools, and outcome measurement methods across the included studies. The absence of gender-specific analyses may also have introduced additional sources of bias and contributed to the heterogeneity observed in the results. Second, only studies published in Chinese or English were included, which may have resulted in selection bias and the exclusion of potentially relevant evidence available in other languages. Third, the present review did not stratify analyses by ADHD subtypes or by participants’ medication use, both of which may influence executive function performance and moderate the effects of physical activity. The included studies also employed diverse executive function assessment tasks, which may have contributed to inconsistencies in effect sizes and increased methodological heterogeneity.

## 5 Conclusion

Current evidence suggests that structured physical activity serves as an important component of non-pharmacological interventions for ADHD. Physical activity exerts significant beneficial effects on executive function and its three core sub-components (inhibitory control, cognitive flexibility, and working memory) in children with ADHD. Physical activity interventions lasting at least six weeks can achieve greater improvements in executive function among children with ADHD. Moderate-to-vigorous physical activity (MVPA) appears to be the most effective intensity threshold for physical activity participation in this population. Open physical activities yield relatively superior improvements in inhibitory control, whereas closed physical activities show better effects on enhancing working memory and cognitive flexibility in children with ADHD. Both single aerobic physical activity and multi-component physical activity effectively improve executive function in children with ADHD; specifically, single aerobic physical activity is more beneficial for inhibitory control and cognitive flexibility, while multi-component physical activity presents a better efficacy in improving working memory. Different types of physical activity can significantly enhance overall executive function in children with ADHD. In terms of inhibitory control, leisure-fitness physical activities demonstrate relatively optimal effects, while ball sports show superior efficacy in improving cognitive flexibility and working memory.

## Data Availability

The original contributions presented in the study are included in the article/**Supplementary Material**, further inquiries can be directed to the corresponding author/s.
