# Supplementary material for: Effects of different physical activity modalities on executive function in children with attention deficit hyperactivity disorder: a systematic review and meta-analysis
Source: Front Psychiatry. 2026 Jun 24;17:1824121. doi: 10.3389/fpsyt.2026.1824121 (PMC13343229; doi:10.3389/fpsyt.2026.1824121)
Supplement: Supplementary file 1 [file DataSheet1.pdf]

NIHR | National Institute for Health and Care Research

PROSPERO
International prospective register of systematic reviews

Effects of Different Physical Activity Modalities on Executive Function in Children with Attention Deficit Hyperactivity Disorder: A systematic review and meta-analysis

Mingyuan Fan, Changchou Chen

Citation 1 change

Mingyuan Fan, Changchou Chen. Effects of Different Physical Activity Modalities on Executive Function in Children with Attention Deficit Hyperactivity Disorder: A systematic review and meta-analysis. PROSPERO 2025 CRD420251080013. Available from https://www.crd.york.ac.uk/PROSPERO/view/CRD420251080013.

Mingyuan Fan, Changchou Chen. Effects of Different Physical Activity Modalities on Executive Function in Children with Attention Deficit Hyperactivity Disorder: A Meta-Analysis. PROSPERO 2025 CRD420251080013. Available from https://www.crd.york.ac.uk/PROSPERO/view/CRD420251080013 [https://www.crd.york.ac.uk/PROSPERO/view/CRD420251080013].	Mingyuan Fan, Changchou Chen. Effects of Different Physical Activity Modalities on Executive Function in Children with Attention Deficit Hyperactivity Disorder: A systematic review and meta-analysis. PROSPERO 2025 CRD420251080013. Available from https://www.crd.york.ac.uk/PROSPERO/view/CRD420251080013 [https://www.crd.york.ac.uk/PROSPERO/view/CRD420251080013].

REVIEW TITLE AND BASIC DETAILS

Review title 1 change

Effects of Different Physical Activity Modalities on Executive Function in Children with Attention Deficit Hyperactivity Disorder: A systematic review and meta-analysis

Effects of Different Physical Activity Modalities on Executive Function in Children with Attention Deficit Hyperactivity Disorder: A Meta-Analysis	Effects of Different Physical Activity Modalities on Executive Function in Children with Attention Deficit Hyperactivity Disorder: A systematic review and meta-analysis

Review type 1 change

None

None

Condition or domain being studied

Attention Deficit Hyperactivity Disorder; Physical activity; Executive Functions

Rationale for the review

In recent years, numerous studies have demonstrated that physical activity can effectively improve and promote executive function in ADHD. However, the forms of physical activity used in these studies vary significantly, including traditional aerobic activities (e.g., running, cycling), aquatic activities, ball games, single-mode aerobic exercise, multicomponent physical activities, and activities of different intensities (e.g., moderate intensity, moderate-to-vigorous intensity). It remains uncertain whether different forms of physical activity produce effects on executive function and to what extent these effects are significant. Therefore, the review aims to conduct a meta-analysis to investigate in depth the effects of different forms of physical activity on the three core subcomponents of executive function (inhibitory control, cognitive flexibility, and working memory) as a whole, in order to offer scientific evidence and guidance for future intervention strategies.

Review objectives

To conduct a meta-analysis to investigate in depth the effects of different forms of physical activity on the three core subcomponents of executive function (inhibitory control, cognitive flexibility, and working memory) as a whole, in order to offer scientific evidence and guidance for future intervention strategies.

Keywords

Attention Deficit Hyperactivity Disorder ADHD; Physical activity; Executive function; Meta-analysis

Country

China

ELIGIBILITY CRITERIA

Population

Included

1)Participants were adolescents aged 6–18 years with a confirmed diagnosis of ADHD;

2)The intervention involved physical activity (e.g., exercise, sports, or physical activities);

3)The control group received standard treatment interventions (e.g., sedentary activities, normal physical activities, or watching videos);

4)The outcome measures included executive function or one of its three core components (inhibitory control, working memory, or cognitive flexibility);

5)The study design was a randomized controlled trial (RCT);

6)There were no significant baseline differences between the control and experimental groups before the intervention;

7)Complete pre- and post-intervention data (means, standard deviations, sample size, etc.) were available;

8)The study was published in Chinese or English.

Excluded

1) Non-RCT studies;

2) Duplicate publications;

3) Reviews, conference abstracts, animal studies, or case reports;

4) Studies that did not report the necessary data, such as only pre- or post-intervention data;

5) Studies with irrelevant topics or inaccessible full texts;

6) Studies not published in Chinese or English.

Intervention(s) or exposure(s)

Included

Physical activity

Comparator(s) or control(s)

Included

PICO tags selected: Waiting list control

1)Watch video

2)No intervention

Study design
Only randomized study types will be included.

Context
Six databases were searched， including CNKI,Wanfang,Web of Science,PubMed,The Cochrane Library, and Embase.

TIMELINE OF THE REVIEW

Date of first submission to PROSPERO
24 June 2025

Review timeline
Start date: 24 June 2025. End date: 24 December 2025.

Date of registration in PROSPERO
24 June 2025

AVAILABILITY OF FULL PROTOCOL

Availability of full protocol
A full protocol has not been written.

SEARCHING AND SCREENING

Search for unpublished studies
Both published and unpublished studies will be sought.

Main sources that will be searched 1 change
The main sources to be searched are *CDSR - The Cochrane Database of Systematic Reviews*, *Embase* and *PubMed*.

Other sources that will be searched
1)Web of Science

2)CNKI

3)Wanfang

The main databases to be searched are CLIB - The Cochrane Library, Embase - Embase via Ovid, Embase.com and PubMed. OTHER IMPORTANT OR SPECIALIST DATABASES THAT WILL BE SEARCHED 1)Web of Science 2)CNKI 3)Wanfang	The main sources to be searched are CDSR - The Cochrane Database of Systematic Reviews, Embase and PubMed. OTHER SOURCES THAT WILL BE SEARCHED 1)Web of Science 2)CNKI 3)Wanfang

Search language restrictions 1 change

The review will only include studies published in Chinese and English.

There are no language restrictions.	The review will only include studies published in Chinese and English.

Search date restrictions 1 change

Databases will be searched for articles published before 20 April 2026, there are no restrictions on search start date.

There are no search date restrictions.	Databases will be searched for articles published before 20 April 2026, there are no restrictions on search start date.

Other methods of identifying studies

Other studies will be identified by: *reference list checking*, *searching conference proceedings* and *searching trial or study registers*.

Link to search strategy 1 change

A full search strategy has been uploaded to PROSPERO. The PDF may be accessed through this link https://www.crd.york.ac.uk/PROSPEROFILES/151cb02223e37f6767fa830893d429da.pdf.

A full search strategy has been uploaded to PROSPERO.	A full search strategy has been uploaded to PROSPERO.

The PDF may be accessed through this link https://www.crd.york.ac.uk/PROSPEROFILES/fea9ccdf50dd6d877f44dd4e8fc3b839.pdf [https://www.crd.york.ac.uk/PROSPEROFILES/fea9ccdf50dd6d877f44dd4e8fc3b839.pdf].	The PDF may be accessed through this link https://www.crd.york.ac.uk/PROSPEROFILES/151cb02223e37f6767fa830893d429da.pdf [https://www.crd.york.ac.uk/PROSPEROFILES/151cb02223e37f6767fa830893d429da.pdf].

Selection process
Studies will be screened independently by at least two people (or person/machine combination) with a process to resolve differences.

Other relevant information about searching and screening
None

DATA COLLECTION PROCESS

Data extraction from published articles and reports
Data will be extracted independently by at least two people (or person/machine combination) with a process to resolve differences.

Authors will be asked to provide any required data not available in published reports.

Study risk of bias or quality assessment
Risk of bias will be assessed using: *Cochrane RoB-2*

Data will be assessed independently by at least two people (or person/machine combination) with a process to resolve differences.

Additional information will be sought from study investigators if required information is unclear or unavailable in the study publications/reports.

Reporting bias assessment
Risk of bias due to missing results will be assessed

Certainty assessment
Certainty of findings will not be assessed

OUTCOMES TO BE ANALYSED

Main outcomes
Executive Function

1)inhibition control

2)working memory

3)cognitive flexibility

Additional outcomes
No

PLANNED DATA SYNTHESIS

Strategy for data synthesis
No formal data synthesis is planned - data will be described but not combined.

CURRENT REVIEW STAGE

Stage of the review at this submission 1 change

Review stage	Started	Completed
Pilot work	✓	
Formal searching/study identification		
Screening search results against inclusion criteria		
Data extraction or receipt of IPD		
Risk of bias/quality assessment		
Data synthesis		

Review status
The review is currently planned or ongoing.

Expand 1 lines ...

Publication of review results
Results of the review will be published in English and Chinese.

REVIEW AFFILIATION, FUNDING AND PEER REVIEW

Review team members
Mr Mingyuan Fan. Shanghai University of Sport. China.

No conflict of interest declared.

Professor Changchou Chen (review guarantor). ORCID: 0000-0002-0553-7013. Shanghai University of Sport. China.

No conflict of interest declared.

Named contact

Mr Mingyuan Fan (2714151523@qq.com). Shanghai University of Sport. China.

Review affiliation

Shanghai University of Sport

Funding source

Review has no specific/external funding but is supported by guarantor/review team (non-commercial) institutions.

Peer review

There has been no peer review of this planned review.

ADDITIONAL INFORMATION

Review conflict of interest

Declared individual interests are recorded under team member details.. No additional interests are recorded for this review.

Medical Subject Headings

Attention Deficit Disorder with Hyperactivity; Child; Executive Function

Revision note 1 change

The manuscript has undergone content revisions.

	The manuscript has undergone content revisions.

SIMILAR REVIEWS

Check for similar records already in PROSPERO

PROSPERO identified a number of existing PROSPERO records that were similar to this one (last check made on 24 June 2025). These are shown below along with the reasons given by that the review team for the reviews being different and/or proceeding.

- Meta-analysis of the effects of physical activity on executive function in children and adolescents with attention deficit hyperactivity disorder [published 13 January 2023] [CRD42023388409]. The review was judged **not to be similar**

- The effect of physical exercise on the executive function of patients with Parkinson's disease: a systematic review and meta-analysis of randomized controlled trials [published 30 March 2024] [CRD42024525647]. The review was judged **not to be similar**
- Effect of physical activity intervention on ADHD executive function : Meta-analysis [published 26 July 2022] [CRD42022346064]. The review was judged **not to be similar**

PROSPERO version history 1 change

- Version 2.0, published 14 May 2026
- Version 1.0, published 24 Jun 2025

Disclaimer

The content of this record displays the information provided by the review team. PROSPERO does not peer review registration records or endorse their content.

PROSPERO accepts and posts the information provided in good faith; responsibility for record content rests with the review team. The guarantor for this record has affirmed that the information provided is truthful and that they understand that deliberate provision of inaccurate information may be construed as scientific misconduct.

PROSPERO does not accept any liability for the content provided in this record or for its use. Readers use the information provided in this record at their own risk.

Any enquiries about the record should be referred to the named review contact
