# Supplementary material for: Effects of different physical activity modalities on executive function in children with attention deficit hyperactivity disorder: a systematic review and meta-analysis
Source: Front Psychiatry. 2026 Jun 24;17:1824121. doi: 10.3389/fpsyt.2026.1824121 (PMC13343229; doi:10.3389/fpsyt.2026.1824121)
Supplement: Supplementary file 2 [file DataSheet2.docx]

PubMed Search Strategy

| Number | Search strategy | Results |
| --- | --- | --- |
| #1 | "Attention Deficit Disorder with Hyperactivity"[Mesh Terms] | 53,207 |
| #2 | (((((((((((((((((((((((ADHD) OR (Attention Deficit Disorders with Hyperactivity)) OR (Attention Deficit Hyperactivity Disorders)) OR (Attention Deficit Hyperactivity Disorder)) OR (Attention Deficit-Hyperactivity Disorder)) OR (Attention Deficit-Hyperactivity Disorders)) OR (Deficit-Hyperactivity Disorder, Attention)) OR (Deficit-Hyperactivity Disorders, Attention)) OR (Disorder, Attention Deficit-Hyperactivity)) OR (Disorders, Attention Deficit-Hyperactivity)) OR (Hyperkinetic Syndrome)) OR (Syndromes, Hyperkinetic)) OR (Attention Deficit Disorder)) OR (Attention Deficit Disorders)) OR (Deficit Disorder, Attention)) OR (Deficit Disorders, Attention)) OR (Disorder, Attention Deficit)) OR (Disorders, Attention Deficit)) OR (Brain Dysfunction, Minimal)) OR (Dysfunction, Minimal Brain)) OR (Minimal Brain Dysfunction)) OR (Inattention)) OR (Impulsivity)) OR (Hyperactivity) | 184,465 |
| #3 | #1 OR #2 | 184,465 |
| #4 | "Exercise"[Mesh Terms] | 671,810 |
| #5 | ((((((((((((((((((((((Exercises) OR (Exercise, Physical)) OR (Exercises, Physical)) OR (Physical Exercise)) OR (Physical Exercises)) OR (Physical Activity)) OR (Activities, Physical)) OR (Activity, Physical)) OR (Physical Activities)) OR (Exercise, Aerobic)) OR (Aerobic Exercise)) OR (Exercises, Aerobic)) OR (Exercise, Isometric)) OR (Exercises, Isometric)) OR (Isometric Exercise)) OR (Isometric Exercises)) OR (Acute Exercise)) OR (Acute Exercises)) OR (Exercise, Acute)) OR (Exercises, Acute)) OR (Exercise Training)) OR (Exercise Trainings)) OR (Training, Exercise) | 922,557 |
| #6 | #4 OR #5 | 922,557 |
| #7 | "Executive Function"[Mesh Terms] | 47,921 |
| #8 | ((((((((Executive Functions) ) OR (Function, Executive)) OR (Functions, Executive)) OR (Executive Control)) OR (Executive Controls)) OR (Cognitive Flexibility)) OR (Inhibitory Control)) OR (Working Memory) | 261,444 |
| #9 | #7 OR #8 | 261,444 |
| #10 | "Child" [Mesh Terms] | 3,560,935 |
| #11 | (Child) OR (Children) | 3,560,935 |
| #12 | #10 OR #11 | 3,560,935 |
| #13 | #3 AND #6 AND #9 AND #12 | \| 280 \| \| --- \| |

The Cochrane Library Search Strategy

| Number | Search strategy | Results |
| --- | --- | --- |
| #1 | MeSH descriptor: [Attention Deficit Disorder with Hyperactivity] explode all trees | 4052 |
| #2 | "Attention Deficit Disorders with Hyperactivity" OR "ADHD" OR "Deficit-Hyperactivity Disorders, Attention" OR "Attention Deficit Hyperactivity Disorders" OR "Disorder, Attention Deficit-Hyperactivity" OR "Deficit-Hyperactivity Disorder, Attention" OR "Disorders, Attention Deficit-Hyperactivity" OR "Hyperkinetic Syndrome" OR "Attention Deficit Hyperactivity Disorder" OR "Syndromes, Hyperkinetic" OR "Attention Deficit-Hyperactivity Disorder" OR "Attention Deficit-Hyperactivity Disorders" OR "Attention Deficit Disorder" OR "Disorders, Attention Deficit" OR "Deficit Disorders, Attention" OR "Attention Deficit Disorders" OR "Disorder, Attention Deficit" OR "Deficit Disorder, Attention" OR "Brain Dysfunction, Minimal" OR "Minimal Brain Dysfunction" OR "Dysfunction, Minimal Brain" OR "Inattention" OR "Impulsivity" OR "Hyperactivity": ti,ab,kw | 12428 |
| #3 | #1 OR #2 | 12428 |
| #4 | MeSH descriptor: [Exercise] explode all trees | 42845 |
| #5 | "Physical Activity" OR "Activity, Physical" OR "Physical Activities" OR "Activities, Physical" OR "Exercise, Physical" OR "Physical Exercise" OR "Physical Exercises" OR "Exercises, Physical" OR "Exercises" OR "Isometric Exercise" OR "Exercise, Isometric" OR "Isometric Exercises" OR "Exercises, Isometric" OR "Aerobic Exercises" OR "Aerobic Exercise" OR "Exercises, Aerobic" OR "Exercise, Aerobic" OR "Active Breaks" OR "Activity Breaks" OR "Acute Exercises" OR "Acute Exercise" OR "Exercise, Acute" OR "Exercises, Acute" OR "Trainings, Exercise" OR "Training, Exercise" OR "Exercise Training" OR "Exercise Trainings": ti,ab,kw | 114000 |
| #6 | #4 OR #5 | 134680 |
| #7 | MeSH descriptor: [Executive Function] explode all trees | 2102 |
| #8 | "Function, Executive" OR "Executive Controls" OR "Executive Functions" OR "Functions, Executive" OR "Executive Control" OR "Inhibitory Control" OR "Cognitive Flexibility" OR "Working Memory": ti,ab,kw | 12645 |
| #9 | #7 OR #8 | 13636 |
| #10 | MeSH descriptor: [Child] explode all trees | 84738 |
| #11 | "Children": ti,ab,kw | 208324 |
| #12 | #10 OR #11 | 208324 |
| #13 | #3 AND #6 AND #9 AND #12 | 113 |

Embase Search Strategy

| Number | Search strategy | Results |
| --- | --- | --- |
| #1 | 'Attention Deficit Hyperactivity Disorder'/exp | 98032 |
| #2 | 'Adhd': ti,ab,kw OR 'Attention Deficit': ti,ab,kw OR 'Attention Deficit': ti,ab,kw OR 'Disruptive Behavior Disorders': ti,ab,kw OR 'Attention Deficit': ti,ab,kw OR 'Disruptive Behaviour Disorders': ti,ab,kw OR 'Attention Deficit Disorder': ti,ab,kw OR 'Attention Deficit Disorder With Hyperactivity': ti,ab,kw OR 'Attention Deficit Hyperactivity Disorder': ti,ab,kw | 76161 |
| #3 | #1 OR #2 | 106114 |
| #4 | 'Executive Function'/exp | 133427 |
| #5 | 'Inhibitory Control'/exp | 3229 |
| #6 | 'Working Memory'/exp | 64415 |
| #7 | 'Cognitive Flexibility'/exp | 5318 |
| #8 | 'Cognitive Inhibition'/exp | 157 |
| #9 | 'Cognitive Control': ti,ab,kw OR 'Executive Control': ti,ab,kw | 21989 |
| #10 | #4 OR #5 OR #6 OR #7 OR #8 OR #9 | 141285 |
| #11 | 'Physical Activity'/exp | 688179 |
| #12 | 'Activity, Physical': ti,ab,kw OR 'Exercise': ti,ab,kw OR 'Exercises': ti,ab,kw OR 'Exercise, Physical': ti,ab,kw OR 'Exercises, Physical': ti,ab,kw OR 'Physical Exercise': ti,ab,kw OR 'Physical Exercises': ti,ab,kw OR 'Physical Activity': ti,ab,kw OR 'Activities, Physical': ti,ab,kw OR 'Physical Activities': ti,ab,kw OR 'Exercise, Aerobic': ti,ab,kw OR 'Aerobic Exercise': ti,ab,kw OR 'Exercises, Aerobic': ti,ab,kw OR 'Exercise, Isometric': ti,ab,kw OR 'Exercises, Isometric': ti,ab,kw OR 'Isometric Exercise': ti,ab,kw OR 'Isometric Exercises': ti,ab,kw OR 'Acute Exercise': ti,ab,kw OR 'Acute Exercises': ti,ab,kw OR 'Exercise, Acute': ti,ab,kw OR 'Exercises, Acute': ti,ab,kw OR 'Exercise Training': ti,ab,kw OR 'Exercise Trainings': ti,ab,kw OR 'Training, Exercise': ti,ab,kw | 804028 |
| #13 | #11 OR #12 | 1224621 |
| #14 | 'Child'/exp | 3893548 |
| #15 | 'Children': ti,ab,kw | 1995493 |
| #16 | #14 OR #15 | 4286168 |
| #17 | #3 AND #10 AND #13 AND #16 | 356 |

Web of Science Search Strategy

| Number | Search strategy | Results |
| --- | --- | --- |
| #1 | TS=(Attention Deficit Disorder with Hyperactivity OR ADHD OR Attention Deficit Disorders with Hyperactivity OR Attention Deficit Hyperactivity Disorders OR Attention Deficit Hyperactivity Disorder OR Attention Deficit-Hyperactivity Disorder OR Attention Deficit-Hyperactivity Disorders OR Deficit-Hyperactivity Disorder, Attention OR Deficit-Hyperactivity Disorders, Attention OR Disorder, Attention Deficit-Hyperactivity OR Disorders, Attention Deficit-Hyperactivity OR Hyperkinetic Syndrome OR Syndromes, Hyperkinetic OR Attention Deficit Disorder OR Attention Deficit Disorders OR Deficit Disorder, Attention OR Deficit Disorders, Attention OR Disorder, Attention Deficit OR Disorders, Attention Deficit OR Brain Dysfunction, Minimal OR Dysfunction, Minimal Brain OR Minimal Brain Dysfunction OR inattention OR impulsivity OR hyperactivity) | 95025 |
| #2 | TS=(Exercise OR Exercises OR Exercise, Physical OR Exercises, Physical OR Physical Exercise OR Physical Exercises OR Physical Activity OR Activities, Physical OR Activity, Physical OR Physical Activities OR Exercise, Aerobic OR Aerobic Exercise OR Exercises, Aerobic OR Exercise, Isometric OR Exercises, Isometric OR Isometric Exercise OR Isometric Exercises OR Acute Exercise OR Acute Exercises OR Exercise, Acute OR Exercises, Acute OR Exercise Training OR Exercise Trainings OR Training, Exercise) | 648178 |
| #3 | TS=(Executive Function OR Executive Functions OR Function, Executive OR Functions, Executive OR Executive Control OR Executive Controls OR Cognitive Flexibility OR Inhibitory Control OR Working Memory) | 265975 |
| #4 | TS=(Child OR Children) | 1323038 |
| #5 | #1 AND #2 AND #3 AND #4 | 377 |
